# Comparison of a fully automatic ARDSNet protocol and a feedback-controlled open lung management concept

**DOI:** 10.1186/cc12035

**Published:** 2013-03-19

**Authors:** A Pomprapa, D Schwaiberger, B Lachmann, S Leonhardt

**Affiliations:** 1 RWTH Aachen University, Aachen, Germany; 2 Charite Berlin, Germany

## Introduction

The aim of this study is to compare two ventilation strategies, the ARDSNet protocol and open lung management, using computer control for 6 hours. The standard therapy for patients with ARDS does typically apply a mechanical ventilator to support breathing. The cost of therapy is high and it requires much attention from physicians to adjust the proper ventilation settings in a timely manner. A closed-loop ventilation concept has therefore been developed and tested with two induced ARDS pigs.

## Methods

The hardware system is composed of a ventilator (Servo 300), a spectrophotometry (CEVOX), a capnography device (CO2SMO+), an electrical impedance tomography device (GOE MF II) and a patient monitor (Sirecust). The software is developed with Labview 7.1. With approval from the ethical committee, two 27 kg pigs were exposed to surfactant depletion with a warm saline washout to induce ARDS (PaO_2_/FiO_2 _<200 mmHg). One pig model was ventilated with an automatic ARDSNet protocol and another was automatically ventilated with open lung management. Blood gas analysis (BGA) was carried out every half an hour.

## Results

Artificial ventilation using the auto ARDSNet protocol successfully stabilized oxygenation, minimized plateau pressure (<30 cmH_2_O), and controlled the pH value for acidosis and alkalosis management. On the other hand, auto open lung management offers a distinctive result of ventilation. A significant improvement of oxygenation and lung compliance was observed within a few breaths after the recruitment maneuvers. Both subjects were ventilated at the same tidal volume of 6 ml/kg and the comparative results of automatic ventilation settings and BGA are provided in Table 1 for every 2 hours.

**Table 1:** Comparative results between auto ARDSNet protocol and auto open lung management

	Before auto ventilation		After 2 hours		After 4 hours		After 6 hours	
				
	Auto ARDSNet	Auto OLM	Auto ARDSNet	Auto OLM	Auto ARDSNet	Auto OLM	Auto ARDSNet	Auto OLM
FiO_2_	1.0	1.0	0.40	0.25	0.40	0.25	0.50	0.25
RR (bpm)	22	40	23	38	23	39	23	35
I:E ratio	1:2	1:1	1:2	1:1	1:2	1:1	1:2	1:1
VT (ml/kg)	6	6	6	6	6	6	6	6
P_plat_/PIP (mmHg)	22	22	18	20.6	15.8	17.7	14.6	15.4
PEEP (cmH_2_O)	5	10	8	15	2	13	10	11
pH	7.34	7.3	7.32	7.52	7.37	7.6	7.38	7.51
pO_2_/FiO_2_ (mmHg)	59	59	180	380.8	155	387.2	122	404
pCO_2_ (mmHg)	52	64	56	39.6	58	31.5	54	41

OLM, open lung management.

## Conclusion

The auto open lung management concept gave much better gas exchange than the auto ARDSNet protocol. These preliminary results showed a necessity to evaluate the two different ventilation strategies. Therefore, further experiments with pig models will be implemented in the near future to obtain results with statistical significance and to ensure the safety of automation in a mechanical ventilation system.

